# Experiences of the homeless accessing an inner-city pharmacy and medical student-run clinic in Johannesburg

**DOI:** 10.4102/hsag.v25i0.1358

**Published:** 2020-04-23

**Authors:** Deanne Johnston, Patricia McInerney, Hilary Thurling

**Affiliations:** 1 Department of Pharmacy and Pharmacology, Faculty of Health Sciences, University of the Witwatersrand, Johannesburg, South Africa; 2 Centre for Health Science Education, Faculty of Health Sciences, University of the Witwatersrand, Johannesburg, South Africa

**Keywords:** homelessness, student-run clinics, accessing healthcare, free clinics, underserved communities

## Abstract

**Background:**

Mental and physical health problems are both contributory factors and drivers of homelessness. Adding to this, the homeless encounter numerous barriers when accessing healthcare services.

**Aim:**

The aim was to determine the experiences of the homeless when accessing healthcare services and the reason why they visit Trinity Health Services (THS).

**Setting:**

Trinity Health Services, a student-run clinic (SRC) based at an inner-city church in Johannesburg, South Africa, provides free acute healthcare services to the homeless.

**Methods:**

This qualitative study comprised three focus group discussions (FGDs) with the homeless who access services provided by the church. Those who had previously visited THS on at least one occasion were invited to participate in FGDs. The FGDs were audio-taped and transcribed verbatim. The data were analysed thematically using Tesch’s eight steps.

**Results:**

Three themes were identified. The first theme, homelessness affecting health, explains how limited access to food, ablution facilities and shelter negatively impact their well-being. This led to the second theme, health needs, where tuberculosis, influenza, sexually transmitted diseases and dental infections were identified as ailments occurring frequently. The need for healthcare services was explicit, yet participants were reluctant when accessing healthcare services as they faced stigma and discrimination. The third theme, THS in addressing health needs, denotes the value of THS in the community it serves where they were treated with compassion and empathy.

**Conclusion:**

The needs of this homeless community as well as the role played by THS were clearly identified. However, THS provides limited services, and integration with existing healthcare services is essential.

## Introduction

The rising levels of unemployment and downward spiral of the South African economy has left many citizens reliant on social grants and facing possible homelessness (Cross et al. 2010; Cross & Seager 2010). The definition of homelessness includes those residing on the street and in communal houses/shelters (Rule-Groenewald et al. 2015). More specifically, homelessness in Johannesburg would expand to include ‘cardboard house under the bridge, occupation of metropolitan open spaces, parks, vacant land, a couple of dirt-stained blankets on the corners of high-rise buildings, occupation of unused buildings’ (Tipple & Speak 2005:343). There are no available statistics confirming the number of homeless in South Africa (Cross & Seager 2010); however, in 2008, it was estimated to be between 100 000 and 200 000 (Rule-Groenewald et al. 2015).

The intersection between homelessness and health is multifaceted. Self-illness or illness of a family member may have been a contributing factor to becoming homeless (Seager & Tamasane 2010). However, poor health is also an outcome of being homeless as the homeless experience higher rates of communicable and non-communicable diseases (Fazel, Geddes & Kushel 2014). Adding to this, the homeless encounter additional problems in accessing healthcare services (Baumstarck, Boyer & Auquier 2015; Trevena, Simpson & Nutbeam 2003).

Student-run clinics (SRCs) provide basic healthcare services to impoverished and underserved communities (Schutte et al. 2015; Simpson & Long 2007). A SRC is an environment where healthcare services are provided by healthcare students under the supervision of licensed healthcare professionals. However, it differs from other clinical exposures as in these clinics the healthcare students are responsible for the management of clinics (Holmqvist et al. 2012; Simpson & Long 2007).

It was found through patient feedback that the homeless were appreciative of the quality of care received at SRCs; however, they raised concerns regarding waiting and operational periods of these clinics (Ellett, Campbell & Gonsalves 2010; Mischell et al. 2017). Feedback from patients and community members assists in identifying the healthcare needs that can be addressed by a SRC (Buchanan & Witlen 2006). Thus, this study was motivated by the need to obtain patient feedback to determine the value of a SRC to the community it serves. The aim of this study was to determine the experiences of the homeless when accessing healthcare services and the reasons for their visiting Trinity Health Services (THS).

## Description of the setting

**FIGURE 1:**
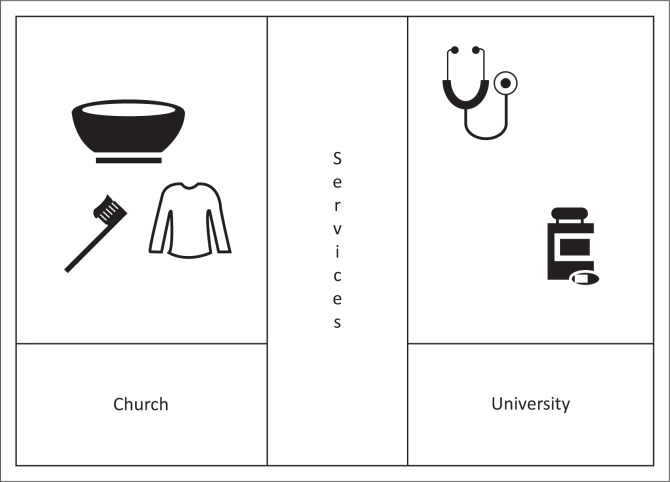
Services provided by the church and the university to the homeless.

Trinity Health Services is a SRC operating on alternate Monday nights from an inner-city church in Braamfontein, Johannesburg. The church provides, apart from meals to the homeless community through a soup kitchen, toiletries (toothbrushes, toothpaste and soap), blankets and second-hand clothing (see Figure 1). The need for healthcare services in the community was recognised by two medical students from the Faculty of Health Sciences, University of the Witwatersrand, Johannesburg, who were volunteering in the soup kitchen (Johnston, Egan & McInerney 2018). Initially, basic first aid services were provided but over time this expanded to more comprehensive acute care. The most common conditions seen and managed at the clinic were related to the respiratory, predominately upper and lower respiratory tract infections, and digestive systems, including dental caries (Johnston, McInerney & Miot 2019). The clinic comprises three consultation rooms and a pharmacy. It is staffed by pharmacy and medical students as well as pharmacists and doctors who work together to provide healthcare services (see Figure 1). Patients in need of further care are referred to nearby public healthcare facilities.

The community served by the clinic has become increasingly dependent on the services provided, yet there is little information describing their experiences when accessing healthcare and the reasons for their attending THS.

Therefore, the research question asked was: *why do the homeless seek help from the clinic and what were their experiences of the services it provides?*

This qualitative study formed part of a larger body of research which aimed to describe the establishment and investigate the various factors affecting the sustainability of THS. A component of this was to establish the value of the clinic from the homeless communities’ perspective and various stakeholders. Stakeholders are groups and/or individuals having a vested interest in THS and were identified as the church, university, pharmacy and medical students, supervising professionals and the homeless community.

## Methodology

Focus group discussions (FGDs) were conducted with the homeless visiting the church to gain an in-depth understanding of their experiences of healthcare services they access. The inclusion criteria were patients who had previously visited THS on at least one occasion and were aged 18 years or older. A convenience sampling method was employed: those meeting the inclusion criteria and were present at the soup kitchen when FGDs were held were invited to participate. The facilitator of FGDs was an experienced qualitative researcher (H.T.). The researcher (D.J.) observed these FGDs.

The facilitator provided a participant-information sheet and explained the purpose of the study to all the participants. The participants signed an informed consent to participate in the FGD and a separate consent was obtained for voice recording. The FGDs were audiotaped and transcribed verbatim. Ethics approval was obtained from the Human Research Ethics Committee of the University of the Witwatersrand, Johannesburg (M170953).

Three FGDs, each with six participants were held, totalling 15 men and three women. The higher number of men who participated is congruent with the demographics of those attending the clinic (Johnston et al. 2019). This is attributed to THS operating after hours and women are more likely to access shelters that close at sunset. The predominant common language spoken by the homeless at the soup kitchen and THS was English. The FGDs varied in duration from 58 min to 75 min.

The facilitator of the FGDs explained to the participants that they could disclose information they were comfortable with sharing and were not expected to reveal personal information relating to their health.

Participants were also requested to keep information discussed in the FGD confidential and not to discuss it with others afterwards. They were asked both primary and probing questions outlined in Table 1.

**TABLE 1:** Primary and probing questions from focus group discussions.

No	Primary question	Probing questions
1.	Please tell me why you attend Trinity Health Services.	-
2.	What are the health needs of the homeless community?	What services are available to the homeless? Where are these located? Are they easily accessible?
3.	How does the clinic serve this community?	How does it differ from other clinics you have visited? How do you communicate your needs to the clinic?
4.	Please describe your experiences of the clinic?	How did you hear about the clinic? How often have you visited the clinic?
5.	What additional services do you or the community need?	How could the care provided to the community be improved? Do you have any other suggestions?

The data were analysed thematically using Tesch’s eight steps (Creswell 2014). The researchers reviewed the audio recordings and transcriptions repeatedly to gain a thorough understanding of the data. Initial codes were induced and grouped into categories. Categories were reviewed to determine the emerging themes.

The source of the statement was confirmed using an annotation system, FGDs were numbered (H1–H3); followed by the participant number (#1–6), the gender of the participant (M: male; F: female) and employment status (E: employed; UE: unemployed).

### Ethical consideration

Participants of the focus group discussions (FGDs) were provided with a participant information sheet, and the facilitator provided an explanation about the purpose of the study. The participants signed an informed consent form to participate in the FGD and a separate consent form for voice recording. The FGDs were audiotaped and transcribed verbatim. Ethical approval was obtained from the Human Research Ethics Committee of the University of the Witwatersrand, Johannesburg (M170953).

## Trustworthiness

Trustworthiness of study was ensured through addressing the ‘credibility’, ‘transferability’, ‘dependability’ and ‘confirmability’ (Guba & Lincoln 1981). The credibility was confirmed through member checking and peer debriefing. The FGDs were conducted by the same facilitator. Before coding commenced transcriptions were checked for accuracy. The coding system and transcription were revised until consensus was reached between researchers. A thick description adds to the ‘transferability’ of the findings to other SRCs and health services the homeless seek. However, THS to our knowledge is the only SRC serving the homeless in South Africa and the findings may be difficult to compare to those SRCs internationally as the services available to these communities differ considerably between countries. The ‘dependability’ refers to consistency of the findings if repeated. Data saturation, defined as the point at which the gathering of more information no longer revealed a new insight, was reached after the third FGD. The ‘confirmability’ denotes the awareness of the researchers bias to ensure neutrality. H.T. and P.M. were not involved in THS. While, D.J. was the responsible pharmacist of Trinity Pharmacy and could not suspend her involvement in THS. She was however aware of her personal bias at all times and the above-mentioned measures were taken to safeguard the trustworthiness of the findings.

## Findings

**FIGURE 2:**
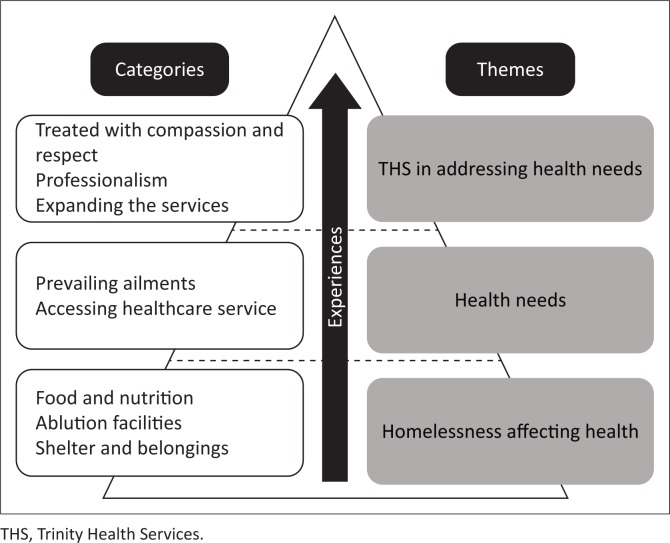
Themes and categories identified.

Three themes emerged from FGDs (see Figure 2). The first theme, *homelessness affecting health*, explains how homelessness affects health. This led to the second theme, *health needs*, referring to the prevailing ailments in the community contributing to their health needs as well as their experiences when accessing existing healthcare services. The third theme, *THS in addressing health needs*, refers to the value of this SRC to the community it serves.

### Homelessness affecting health

There were three categories contributing to this theme: food and nutrition, ablution facilities, as well as shelter and belongings. These categories elucidate the link between homelessness and health. The homeless frequent soup kitchens for food or may resort to eating from dustbins. There are few ablution facilities in the vicinity and they cannot afford toiletries, resulting in poor hygiene practices. Many of the homeless reside on the streets as they cannot afford to stay in a shelter. Exposed to the cold wet weather, they frequently become sick. Furthermore, their belongings, such as important documents, medication, clothes and blankets, were often stolen.

#### Food and nutrition

The participants visited the daily soup kitchens provided by the church or other facilities in the surrounding areas. However, many reported to have eaten from dustbins when necessary. Some explained the pain they experienced from hunger:

‘You never know how hunger is, how painful it is until you get hungry yourself.’ (H2, #2, M, UE)

Mixed views were expressed regarding the food they ate, most often soup, and how often they were provided with meals. However, all agreed that their diets lacked nutritional value as indicated in reply to this question asked by a participant:

‘Where do you get a balanced meal if you have to beg for food?’ (H1, #4, M, UE)

Furthermore, those taking medication were often instructed to take it with food. This may lead to non-compliance to medical treatment if they have nothing to eat.

#### Ablution facilities

The homeless use public ablution facilities to wash themselves and their clothes. Because they have to wait in long queues to use ablution facilities, they found:

‘It’s very difficult on the street to get a bath every day.’ (H1, #4, M, UE)

In addition, there is only cold water, and they need to purchase their own toiletries and soap, which they cannot afford.

#### Shelter and belongings

Some participants slept in a shelter but were required to pay a fee. Many of the homeless cannot afford this fee, leaving them with no choice but to sleep on the pavements. This, in turn, affects their health as they are more prone to influenza and infections:

‘When you sleep on top of a pavement … if it rains, you normally (have) some cardboard underneath and then you put a plastic on top … the rain overflows underneath the cardboard, and it’s just there before you get wet. And then the thing is we catch “flu” or coldness during that process … there is only one part that is being affected, and that is the back bone (referring to the spine). So it absorbs or it catches that coldness, and then after that what happens, you’re going to get a blocked chest.’ (H3, #2, M, UE)

They face many difficulties when finding shelter and keeping their belongings safe, such as identification documents and medication:

‘The basic thing is somewhere to sleep and where my things such as my CV can be kept safe … You can’t keep a prescription of medication for more than 3 days without losing it.’ (H1, #1, M, UE)

### Health needs

The theme ‘health needs’ was developed from two categories: prevailing ailments and accessing healthcare services. The participants described frequently occurring ailments such as influenza, tuberculosis, sexually transmitted diseases and dental infections that necessitated treatment. The participants then explained their experiences when accessing healthcare facilities and the barriers encountered.

#### Prevailing ailments

Tuberculosis and drug/alcohol addiction were identified by the participants as the prevalent conditions amongst the homeless:

‘The majority (of the homeless) are doing drugs and you have got to watch out they are bringing in TB [*tuberculosis*] and viruses and they smoke from each other’s lips (as they share cigarettes), so TB it’s a high risk on the streets.’ (H2, #6, M, UE)

This participant described the need for information about sexually transmitted infections and, in particular, using adequate protection to reduce their transmission:

‘I will wish that it can be also more of a counselling in terms of sexually transmitted disease … because in the street I don’t think they exercise protection.’ (H1, #5, M, UE)

The need for healthcare was evident especially during the winter months when those residing on the pavements were exposed to extreme cold. Most participants stated that they had had flu, and one participant explained that flu vaccines were required to provide protection against influenza:

‘During winter it’s whereby we experience a high number of death rates on the street, due to the fact that people, they don’t know their health state.’ (H3, #4, M, UE)

The necessity for dental service was evident. Participants explained that there were relatively few facilities offering this service, and adding to this many of the homeless cannot afford toothpaste and toothbrushes:

‘Since I was born I have never been attended to by a dentist.’ (H1, #6, M, UE)

‘My brothers and sisters are losing … their teeth, based on certain food they eat and not being able to have toothpaste and toothbrush to keep them clean.’ (H3, #2, M, UE)

Diseases that the homeless present with were also described as ‘hidden sicknesses’ (H3, #6, M, UE), referring to people not knowing about their condition, and therefore ‘… they fail to come here to approach the doctors for their health issues’ (H3, #4, M, UE).

#### Accessing healthcare services

The need for healthcare services was explicit, yet participants were reluctant to access these (government clinics) services as they felt discriminated against:

‘I was afraid to go to the public hospital because I know how they treat people.’ (H1, #5, M, UE)

‘They (clinics and hospitals) are avoiding homeless people to give them such care, or to give them support in their rights (to healthcare).’ (H3, #2, M, UE)

The stigma and discrimination the homeless face is contextualised in these two questions that a participant was frequently asked by healthcare professionals:

‘Why are you not bathing? and

‘Why are you smelling?’ (H1, #1, M, UE)

Furthermore, they were blamed for the illnesses they encounter:

‘It’s like when you’re not clean or when you’re not formally dressed or not smartly dressed, they’ll be like you went to seek the sickness …’ (H3, #2, M, UE)

This participant explains the distrust of the homeless by healthcare professionals. For example, when reporting that medication was stolen and needed to be replaced:

‘I’m HIV [*human immunodeficiency virus*] positive, they [*healthcare professionals*] don’t trust, like the medication I’m taking is for 30 days, they don’t trust me. If I get lost one of the, my ARVs [*antiretrovirals*] it’s like I’ve sold it, you see.’ (H3, #2, M, UE)

Providing identification documents and residential addresses, traveling costs, operational hours of clinics and high volumes of patients were also described as limiting factors that impede the homeless from accessing healthcare services. The participants explained that their identification documents were often stolen and without these they cannot access public healthcare services:

‘Almost 99% of us here have got … no IDs [*Identification documents*], they are stolen at night.’ (H1, #1, M, UE)

### Trinity Health Services in addressing health needs

The homeless are welcomed by the church through the soup kitchen and healthcare services provided. This participant explained that the church felt like home and the people who access these services regularly have formed a community:

‘… [*I*]t’s more like our home, our community …’ (H3, #3, M, UE)

Trinity Health Services was appreciated by the participants for the medical and pharmaceutical facilities provided. The value of the clinic to this community extends beyond these services forming the first two categories in this theme: treated with compassion, and respect and professionalism. The third category, expanding the services, describes unmet needs of the community and suggestions for the future services.

#### Treated with respect and compassion

The participants described the way they were treated in contrast to the stigma they so often faced when accessing other healthcare services:

‘[*Y*]ou have value, you’re understood, you’re just someone.’ (H3, #4, M, UE)

They felt respected when visiting the clinic, which positively affected their health:

‘The respect that they are giving us as homeless people is one of the most important things in terms of it’s one of the things that encourages us.’ (H1, #5, M, UE)

They also referred to the ‘love and compassion’ (H1, #4, M, UE) experienced at the clinic, which in turn motivated them to return to the clinic:

‘Some other time you just wish that you can be sick forever so that you get that attention.’ (H2, #2, M, UE)

#### Professionalism

The participants referred to the professionalism of those providing the services at THS. The privacy experienced during consultations and relationships formed between healthcare providers and patients were key components that contributed to this category.

The participants were aware that THS is a SRC and felt that this was to their advantage because the pharmacy and medical students spent more time with them and there was a ‘double check’ in place as the students presented to a supervisor:

‘The check-ups are being done from A to Z and they even check or they double check … there are doctors that are doing their (clinical) practicals and then there are superiors on top of them, so what I love about that is that they [the pharmacy and medical students] are the practical ones and are giving the superiors the information, and the superior one double checks.’ (H3, #2, M, UE)

A relationship was established between the patient and the pharmacy and medical students, and the patient felt comfortable in disclosing the necessary information:

‘They [*the pharmacy and medical students*] give you enough time to even ask you what other things are bothering you … by asking me what other things are bothering me … I was able to open up … I will really say that for them to come to where we are, it’s one of the best advantages they are giving us.’ (H1, #5, M, UE)

#### Expanding the services

The clinic is open on specific evenings each month and offers limited services. Therefore, patients were frequently referred to public healthcare facilities for further care. The following comment explains the disadvantages of the referral system, as they return to the healthcare services where they are discriminated against:

‘Most of the times it’s unfortunate that they only have to take you to refer you somewhere else to these people whom we are complaining about.’ (H1, #1, M, UE)

Participants suggested that THS must be opened more frequently and needed to expand the services offered to include dentistry, social and psychological services, as well as health promotion:

‘The only thing that I feel like it can be improved is [*to open*] two times or three times a week at least, and then as well as a dentist.’ (H3, #2, M, UE)

‘A support group by which we are sharing our health circumstances … giving courage to one another.’ (H3, #2, M, UE)

## Discussion

This study described the experiences of the homeless accessing care from a free SRC based at an inner-city church. However, their experiences extended beyond THS and explained the connection between being homeless and health as well as the need for such a clinic. Three themes emerged: homelessness affecting health, health needs and the role of THS in addressing health needs. Limited access to food, ablution facilities and shelter negatively affected the health of the homeless. Adding to this, the stigma and discrimination they faced at public health services adversely affected their mental and psychological health. They explained the challenges faced when accessing public healthcare services and the gap which THS has filled.

This study reports on the experiences of participants when accessing healthcare services. Although it may be difficult to confirm the accuracy of their descriptions, other studies have also reported the discrimination that the homeless face when accessing healthcare services in South Africa (Moyo, Patel & Ross 2015; Seager & Tamasane 2010; Wentzel & Voce 2012). Their negative experiences whilst accessing healthcare services could lead to non-compliance with treatment regimens, especially in the case of HIV-positive patients as described in these findings. This could result in treatment failure and the virus developing possible resistance to the medication.

The participants appreciated the services as well as the respect and compassion provided by the SRC. In addition, the participants described returning to the clinic when they were sick or waiting till the clinic was open again for seeking help. Student-run clinics have been shown to provide quality healthcare services that lead to positive patient outcomes (Berman et al. 2012; Butala et al. 2012; Der et al. 2001; Gorrindo et al. 2014; Hsu et al. 2003; Liberman et al. 2011; Lough, Ebbert & McLeod 2011; Ryskina, Meah & Thomas 2009; Spector, Alpert & Karam-Hage 2007; Zucker et al. 2011). Although studies have reported positive patient outcomes and feedback, it is important to note that most SRCs provide services to vulnerable communities and need to ensure the needs of the patients are always prioritised (Buchanan & Witlen 2006).

Trinity Health Services is striving towards becoming a patient-centred clinic. The three core factors of patient-centred care are: patients, including their family; healthcare professionals; and a healthcare environment (Epstein & Street 2011) that ensures the care rendered is ‘respectful of and responsive to individual patient preferences, needs, and values, and ensuring that patient values guide all clinical decisions’ (Committee on Quality of Health Care in America 2001:3). Understanding the healthcare needs of the community THS serves and how this integrates with their social circumstances are imperative for strengthening and expanding the services. Future studies need to explore suitable means to measure the interventions the clinic takes in becoming a patient-centred clinic.

There were several instances when participants mentioned their referral to other clinics and hospitals for further care. An established relationship between the clinic and neighbouring facilities is needed to expedite the referral process. Gelberg, Andersen and Leake (2000) found that the homeless were more likely to seek help of healthcare services if they felt the need. Thus, the referral letter the patients receive could prompt them to seek further care. However, more research is needed to determine whether referred patients visit these facilities and the factors influencing their decision to access further care.

The gap, which THS fills, is clearly elucidated. However, the community calls for more assistance, perhaps beyond the scope and the capacity of the clinic. The clinic plans to incorporate dental services. There is also an urgent need for social and psychological services as well as skills development. The church and the university must consider including additional partnerships with government and non-government organisations extending beyond the scope of this SRC.

## Conclusion

This homeless community described an urgent need for healthcare and the challenges faced when accessing the existing services. The compassion and empathy received by patients at THS created an environment conducive to healing where patients felt at ease, and trusting relations with healthcare providers were formed. However, THS provides limited services; thus, integration with existing healthcare and other services is essential to meet the needs of the community.
